# Improving Spasticity by Using Botulin Toxin: An Overview Focusing on Combined Approaches

**DOI:** 10.3390/brainsci14070631

**Published:** 2024-06-24

**Authors:** Loredana Raciti, Gianfranco Raciti, Antonio Ammendolia, Alessandro de Sire, Maria Pia Onesta, Rocco Salvatore Calabrò

**Affiliations:** 1 Unità Spinale Unipolare, AO Cannizzaro, 98102 Catania, Italy; loredana.raciti79@gmail.com (L.R.); mariapia.onesta@gmail.com (M.P.O.); 2 Department of Medical and Surgical Sciences, Magna Græcia University, 88100 Catanzaro, Italy; gianfranco.raciti@gmail.com (G.R.); ammendolia@unicz.it (A.A.); alessandro.desire@unicz.it (A.d.S.); 3 IRCCS Centro Neurolesi Bonino Pulejo, 98121 Messina, Italy

**Keywords:** botulin toxin, spasticity, antispastics, robot-assisted motor training, Lokomat

## Abstract

Spasticity is a very common sign in the neurological field. It can be defined as “a motor disorder marked by a velocity-dependent increase in muscle tone or tonic stretch reflexes” associated with hypertonia. It leads to a high risk of limb deformities and pain that prejudices residual motor function, impairing quality of life”. The treatment of spasticity depends on its severity and its location and, in general, it is based on rehabilitation, oral therapies (the gamma-aminobutyric acid b agonist baclofen) and injectable medications (i.e., botulin toxins, acting on polysynaptic reflex mechanisms). The botulin toxin type A (BoNT-A) injection has been effectively used to improve different types of spasticity. However, when BoNT-A is not sufficient, a combination of nonpharmacological approaches could be attempted. Therefore, additional intervention, such as conventional physical therapy by itself or further combined with robotic gait training, may be needed. Indeed, it has been shown that combination of BoNT-A and robotics has a positive effect on activity level and upper limb function in patients with stroke, including those in the chronic phase. The aim of this review is to evaluate the efficacy of pharmacological or nonpharmacological treatment in combination with BoNT-A injections on spasticity. The combined therapy of BoNT with conventional or adjunct activities or robot-assisted training, especially with end-effectors, is a valid tool to improve patients’ performance and outcomes. The combined strategies might rise the toxin’s effect, lowering its dosages of botulinum and reducing side effects and costs.

## 1. Introduction

Spasticity is an increased muscle tone due to the impairment of the upper motor neuron with exaggerated deep tendon reflexes [1] with damage of synaptic plasticity at the spinal cord [2] after a brain injury. Patients with stroke sequalae as paresis may be associated with spasticity. However, spasticity could be the symptom of several diseases, such as spinal cord injury, multiple sclerosis, parkinsonian syndromes, cerebral palsy, dystonia (presenting as muscle overactivity called spastic dystonia), spasms, and spastic co-contractions.

In stroke patients, spasticity could develop after about 3 months in 19% of patients or after 12 months in 38% of patients after the acute event [3,4]. About 65% to 85% can walk within the first 6 months after stroke, usually with limitations in activities of daily living and impairment of their quality of life [4]. Consequently, spasticity is widely disabling due to increasing joint contractures and pain, the limitation of the range of motion (ROM) of the joints, limiting walking (when spasticity of the lower limbs is developed), or the limitation of activities of daily living, such as dressing, hygiene, or feeding (even when upper limbs are involved) [5].

Botulinum neurotoxins (BoNTs) are proteins generated by the spore-forming bacteria clostridium, including botulinum [6,7,8], causing flaccid paralysis. The first therapeutic indication of this toxin was for strabismus in 1977, involving the infiltration of the extra-ocular muscles [7]. Since then, the botulinum toxin has been widely applied in several diseases, and innovative formulations have been approved on the market, including (and mainly) for spasticity treatment. The seven available types of BoNTs, from A to G [9], differ structurally and in toxo-pharmacological properties. The classification is based on the serological typing of the toxins and their variation in amino acid sequence [10].

BoNTs inhibit the neurotransmitter release of ACh from the cholinergic nerve terminals of the skeletal muscles [11,12,13,14,15] and influence neurotransmission at chemical synapses in the peripheral and central nervous system, whose mechanism of action is critical to nociception [16]. Conventional BoNTs used in clinical practice are three types of A1 serotype, including onabotulinumtoxinA (onaBoNT, Botox), AbobotulinumtoxinA (aboBoNT), and IncobotulinumtoxinA (incoBoNT, Xeomin), and a serotype B, RimabotulinumtoxinB (rimaBoNT, Myobloc/Nuerobloc) [17,18] (Table 1).

These formulations are not identical or equivalent, and the therapeutic effects depend on the injection volume, toxin concentration, and dose [17,19]. Botulinum toxin type B (Myobloc^®^ in the United States, NeuroBloc^®^ in Europe) is available in a ready-to-use solution for injection in three vials, 2500 U, 5000 U, and 10,000 U. Currently, efficient and safe therapeutic application is the focus of treating of hyperhidrosis, cervical dystonia, and sialorrhea. Side effects have been described, such as dysphagia and dry mouth, as well as a higher affinity for autonomic nerve endings than BoNT-A [19]; however, lower hyposthenia, as with the BoNT-A type, has been reported. The efficacy could be evaluated after one week and maintained over thirteen weeks [19].

Usually, the effect of BoNTs type A (BoNT-A) starts 2–4 days after injection and raises the maximum effect at 3 weeks [20].

The aim of this paper is to summarize the use of BoNT-A in the management of spasticity, focusing on combined approaches. In particular, after having better specified the potential role of BoNT-A in reducing spasticity, we focused on the combined approaches, both pharmacological and not (i.e., BoNT-A plus orthosis, physiotherapy, shockwaves, electrostimulation, and robotics).

## 2. Botulin Toxin and Spasticity

Several open and placebo-controlled studies have reported the efficacy of local botulinum toxin injections in reducing spasticity and empathizing its easy use and safety [5,21]. Consequently, the toxin has been approved for many conditions, including neurological diseases, cosmetics, urologists for bladder injections, and pain [22]. By now, BoNT-A represents the gold standard therapy for focal spasticity [21,23], presenting a clinically immediately significant effect with good tolerance. In 2015, the effect of botulinum toxin A on upper spasticity compared to a placebo was studied in patients with traumatic brain injury (TBI) [24]. TBI patients were treated with abobotulinumtoxinA (500 U or 1000 U), and nine were given a placebo. After four weeks from the injection, patients treated with the toxin showed an improvement in the angle of catch (XV3 of the TS), finger (+35 degree), elbow (+22 degree), and wrist (+12 degree) flexors, obtaining an increase of at least 5 degrees active range of movement without a clear statistically significant result. Naumann et al. demonstrated the efficacy and safety of BoNT-A after a long administration period [25]. The same results were obtained in studies on the spasticity of patients post-stroke who reported the positive impact of repeated administration of BoNT-A on spasticity and the maintenance of patients’ functions [26]. On the other hand, few studies have reported limited efficacy of BoNT-A therapy for lower limb spasticity and of combined therapy with rehabilitation [27]. This may be likely related to the short lifetime of two to three months, leading to a brief reinjection interval that is considered a burden for patients [28,29].

Another reported side effect of BoNT injections is the development of neutralizing antibodies of BoNT-A in about 44% of injected patients, which compromises the biological effects of the treatment, causing a reduced response to the drug. This could require a progressive rise in the amount of BoNT dosage over time to enhance and achieve the clinical effect and the healthcare budget [25,30]. Consequently, several rehabilitation techniques have been added to the BoNT injection [31], and studies on the efficacy of combination therapy of BoNT and non-pharmacologic treatments have been reported, showing improvement and maintenance on clinical assessment targets [32,33]. Quite a lot of studies have shown that physical therapy after focal BoNT-A injection improves the neurotoxin effect [34]. In 2018, Hara et al., for the first time ever, found that the combined therapy of BoNT-A injection and rehabilitation for post-stroke patients with spasticity enhanced and preserved the improvement of spasticity, as well as the lower limb motor functions in post-stroke patients [35]. However, some studies have also highlighted that the real benefit is doubtful [36].

## 3. Toxins plus Other Antispastics

Spasticity can benefit from several medications that act either on the CNS or on the muscles directly. The various pharmacological treatments used to reduce muscle tone, orally, by injectable administration, or through an intrathecal pump, showed controversial efficacy (Table 2).

To decrease the overall level of spasticity, oral or intrathecal therapy may be combined with local injections of BoNT to address focal problems related to spasticity [37]. Due to the short duration of the effects, oral therapy is administered about every 4–6 h. Usually, a single dose at bedtime is preferred to control nighttime spasms and reduce daily drowsiness. Indeed, the side effects, such as sedation and weakness, limited their use. Baclofen and tizanidine are the most used. Tizanidine has been recommended for patients with chronic stroke because of the potential negative impact of benzodiazepines on brain plasticity in the post-stroke recovery period [38]. The use of gabapentin and pregabalin as adjunct therapies has been finalized for central neuropathic pain. Recently, phase III clinical trials in patients with spasticity due to multiple sclerosis have shown the efficacy of cannabis derivatives to treat muscle hyperactivity, but not for post-stroke spasticity [39]. An intrathecal baclofen pump has a direct action on the spinal cord because of the straight infusion of GABA agonist in the spinal cord. This procedure has been used particularly for spasticity of the lower limbs and the trunk. However, severe spasticity does not respond to this treatment in decreasing muscle hyperactivity. For this reason, botulinum toxins are used for the treatment of localized spasticity of the upper limbs. This approach increases patients’ abilities to actively mobilize their upper and lower limbs and improve their autonomy (e.g., self-care, walking) [40,41,42]. However, adverse events have been described [43].

## 4. Toxins plus Nonpharmacological Approaches

Nonpharmacologic adjunct therapy (AT) may improve the benefits of BoNT-A, but the results are controversial, with a gap in the expert consensus on the use of AT after the injections. A recent systematic review has been conducted on the overall literature results. Generally, all experts recommended adjunct therapies after BoNT-A based on the goals of the treatment, unless the treatment targets pain or when the participation of the patient is impossible.

### 4.1. Toxins and Orthosis

For example, orthosis combined with BoNT-A injection has been shown to be effective for the treatment of pes varus and equinovarus foot, clonus, and walking [44] in patients with post-stroke paresis related to triceps sura spasticity. Orthosis has been used as an ankle–foot aid to avoid muscle rigidity and joint deformity and to recover walking and motor skills [45] such as walking speed and increased peak ankle dorsiflexion during both the stance and swing phases [46]. The literature is scarce on the number of trials that could show a BoNT-A effect on velocity improvement [47]. Only a recent systematic review has shown an improvement in gait velocity of a 0.044 m/s increase (with an effect size of 0.193). The authors also found that the combined therapy of BoNT and rehabilitation gradually distributed the dosage amount of BoNT-A into other muscles over the treatment course. Teasell et al. examined the rehabilitation management of post-stroke patients and reported that proper management and training for spasticity was effective for the improvement of motor function in post-stroke patients, even at 6 months or more after onset [48].

### 4.2. Toxin, Electrical Stimulation, and Physical Therapy

The long-term effect of BoNT-A has been studied by using electrical stimulation (ES), which rushes the binding and internalization, as well as the translocation, of BoNT-A at the motor nerve terminals in animal models just a few minutes after BoNT injection [49]. Several studies have also been conducted in humans. However, a recent review has reported that all studies were not in accordance with stimulation procedures [34]. Controversial opinions have been reported in a few reviews [49,50,51]. A recent review reported that a consensus could not be reached on the use of ES (71%), and the use of other stimulations, such as muscle vibrations and shockwave therapy (consensus by 100% and 84%), was not recommended for spasticity. The optimal adjunctive therapy to be used depends on the treatment goals. However, the use of continuous posture techniques, such as taping and casting, after BTIs is recommended to improve the passive range of motion or spasticity, but not to enhance active functions. The continuous posture should be applied for 1 to 2 weeks after BTIs; meanwhile, adjunct active therapy has been recommended for at least 3 h per week, even though the optimal total duration is unknown. Moreover, the use of low-intensity manual stretching, as well as the use of K-taping or compression sleeves, are not recommended [32].

### 4.3. Toxin and Extracorporeal Shockwave Therapy

Another adjunct therapy suggested to improve post-stroke spasticity is extracorporeal shockwave therapy (ESWT) [52,53,54], which consists of focal pressure pulses with about 1000 impulses on the spastic muscle. Even though, recently, the experts did not recommend the use of ESWT, some studies reported the safety and effectiveness of 500 non-focused pulses of low-energy ESW, showing a statistically significant improvement in hypertonic muscles for several weeks [53,54,55]. Manganotti and Amelio showed the safety and effectiveness of one ESWT session in the reduction of wrist and finger flexor spasticity in stroke patients persisting for at least 12 weeks, without adverse events [54]. Moreover, a single ESWT stimulation was used for children affected by cerebral palsy with spastic equinus foot [56]. However, the reported overall study results in chronic stroke or multiple sclerosis patients could be extended to other central pathology manifestations with spasticity [32].

### 4.4. Toxin and Robot-Assisted Motor Training

The training that best showed an improvement in motor impairment was task-oriented training [57] simulating real-life tasks. Two examples of this training are robot-assisted training (RAT), which is an individualized, intensive training with variable sensorimotor feedback and kinesthesia input for motor relearning [58,59,60], and mirror therapy, a practical intervention to improve UE function [61] using a mirror placed between two arms. Therefore, the inverse reflection of the moving unimpaired arm builds a visual illusion increasing the impaired arm’s ability to move [61,62]. RAT and mirror therapy induce neuroplastic changes, enhancing ipsilateral damaged hemisphere cortical activation [61,63], even though it is associated with the mirror neuron system [64]. It has been shown that a combined therapy of BoNT-A and RAT reduces spasticity and improves muscle strength in patients with stroke more than RT alone [65,66] or BoNT-A combined with conventional treatment [67].

The treatment of impaired walking in post-stroke patients has been widely studied, with several rehabilitation conventional protocols with or without adjunct therapies, as previously reported (ESW, ES, taping, and so on). Robot-assisted gait training (RAGT) has been recently introduced in the training sessions of stroke patients but also in patients with SCI and Parkinson’s disease, with remarkable results [63,68,69]. The advantage of RAGT is the increase in the afferent feedback associated with normal locomotion and the capability to induce plasticity in the involved motor centers [69] through early, intensive, repetitive, and contextual training facilitating the relearning of motor function [69,70]. As far as this, it has been shown that RAT is regarded as an effective and reliable method for the delivery of highly repetitive training that is needed to trigger neuroplasticity [69]. Lokomat training is considered a promising method to restore functional walking and promote locomotor ability, which might enable people to enhance their level of physical activity and improve their activities of daily living [70]. In fact, it has been found that the Lokomat leads to changes in several spinal reflex pathways and changes to the spinal reflex circuitries interacting with the autonomic system as inter-limb coordination [71], with a re-emergence of physiological phase modulation of the soleus H-reflex during gait [65,66].

RAGT includes conventional overground walking training and body weight-supported treadmill training (BWSTT) [72]. BWSTT allows for the early initiation of gait training, the integration of weight-bearing activities, stepping, and balance using both a task-specific approach and symmetrical gait arrangement [72]. Based on the severity of hemiplegia/hemiparesis, we could choose the kind of functional trial using exoskeletons (for severe hemiplegia: Lokomat [73], BLEEX [74], HAL [75], and LOPES [76]) or end-effectors (for mild to moderate motor impairment: gait-trainer, G-EOSystem, and haptic walker [57,58,77,78,79,80,81]). Recently, a systematic review has shown that RAGT raised the recovery of independent walking in stroke patients with severe motor impairment. Therefore, it has been suggested as an “add on” treatment [82] and, rarely, as a substitution for conventional rehabilitation, either in the early phase or in chronic patients [83,84,85]. However, there is still controversy surrounding which different phases of stroke are the best window to start RAGT, how to perform the best rehab protocol, and whose patients may benefit from a specific robotic device [86,87,88]. The American Heart Association and the American Stroke Association have suggested that combined therapy of electro-medical devices and conventional therapy within the first three to six months of acute/subacute strokes is more effective than traditional therapy alone. Therefore, robot-assisted gait training with a treadmill, visual/acoustic cues, training with virtual reality, exoskeletons (Lokomat), and end-effectors (GT3) are indicated subsequently to the acute event in stroke patients and the impairment of walking.

Picelli et al. have demonstrated that the combined treatment with BoNT-A and robotic gait training with a Lokomat versus BoNT-A injection alone in individuals in the chronic post-stroke phase improved the 6 min walk test, but showed no improvement on the modified Ashworth scale or the Tardieu scale [89]. On the other hand, Erbil et al. observed the efficacy of RAT (RoboGait^®^) plus physical therapy and previously injected BoNT-A compared with physical therapy alone in the chronic post-stroke phase, showing statistically significant improvements in spasticity, balance, and gait functions after treatment. [90]. Recently, Cotinat supported the efficacy of Lokomat plus toxins in the improvement of walking in individuals with chronic post-stroke compared with conventional physiotherapy [91].

Important limitations in activities of daily living (ADL) have also been shown to be related to upper extremity dysfunction in 30–66% of stroke patients. Focal therapy using botulinum toxin injections has led to substantial improvement, which could be potentiated by botulinum combination therapies, such as electrical stimulation therapy, repeated transcranial magnetic stimulation therapy, and RAT [59,60,61,71,92].

The single-joint hybrid assistive limb exoskeleton (HAL-SJ) has shown efficacy in assisting the flexion/extension movement of the elbow joint [93,94,95,96]. The robot is based on the principle of paired associative stimulation (PAS), relating the desire to move and the actual movement evoked proprioceptive feedback, promoting brain plasticity [93,94,95]. Repetitive median nerve stimulation, combined with transcranial magnetic stimulation (TMS) over the contralateral motor cortex, induces plastic changes in excitability and long-term potentiation in the brain, which is applicable to learning and memory [2]. However, the HAL-SJ has shown an immediate improvement in the active ROM of the elbow joint, even after a period of about 1 year from the BoNT injection, but only in the proximal region of the upper limb [95]. Despite these limitations, the combination therapy of conventional rehabilitation with robot-assisted training has been shown to be superior to BoNT injections with conventional rehabilitation alone [97,98,99].

## 5. Discussion

Spasticity is a common sign of neurological disorders, and healthcare professionals involved in neurorehabilitation have to deal with it in their clinical practice daily. Indeed, spasticity is often associated with many of the symptoms and signs belonging to upper motor neuron syndrome, such as spasms, clonus, dystonia, and spastic co-contractions (i.e., a simultaneous contraction of the agonist and antagonist muscles resulting in alterations of movements and dexterity), as well as abnormal reflex responses, muscle fatigue and weakness, up to muscle nonuse atrophy. Therefore, the proper treatment of spasticity is a main goal in neurorehabilitation in order to improve functional outcomes. The currently available interventions to treat spasticity include preventive measures (like stretching and posturing), conventional therapeutic interventions (such as physiotherapy, occupational therapy, and hydrotherapy), physical therapies (like muscle vibration and electrical stimulation), oral medications (such as baclofen, benzodiazepines, gabapentin and dantrolene), injectable medications (such as botulinum toxins and phenol), and the use of orthotics and surgical interventions.

BoNT injections represent the gold standard therapy of focal spasticity not responding to nonpharmacological therapy due to the direct central action, which is reducing muscle tone [5,6]. BoNT should be injected into key muscles to reduce focal spasticity and modify synergic movements and/or co-contraction and spasms. When BoNT-A is not sufficient to manage spasticity and related symptoms, it could be useful to move toward other therapeutic options, including robotics. It has been shown that BoNT could recover the functional muscle of the limb when it is combined with therapies that influence synaptic plasticity, such as RAT [70]. Indeed, to enhance the effectiveness of BoNT injections, optimal adjunctive therapy based on the treatment goals could be used, and then specific and personalized physiotherapy programs should follow. Because of the lack of approved standardized protocols for central pathologies, recent recommendations on adjunct therapies on the effect of BoNT in chronic stroke or multiple sclerosis patients could be extended to the other central pathologies manifesting with spasticity [32]. At this time, no adverse events have been reported in all combined therapies, besides the well-known adverse events of the sole therapies themselves.

Allart et al. [32] declared that no consensus has been reached for the use of ES, shockwave therapy (ESWT), or muscle vibrations after BoNTs. The experts did not recommend the use of ESWT, but some studies reported a statistically significant improvement in hypertonic muscles for several weeks with 500 non-focused pulses of low-energy ESW [53,54,55].

The various oral pharmacological treatments used to reduce muscle tone showed controversial efficacy. For instance, due to the short duration of the effects, oral therapy is administered about every 4–6 h with a high incidence of side effects, such as sedation and weakness, that limit their use.

In patients with spasticity due to multiple sclerosis, treatment with cannabis derivatives has been shown efficient in reducing muscle hyperactivity, but not for post-stroke spasticity [39].

An intrathecal baclofen pump directly infuses a GABA agonist into the spinal cord, and it has been specifically used for spasticity of the lower limbs and the trunk. However, it is not efficient for severe spasticity.

On the other hand, the use of continuous posture techniques, such as taping and casting, after BTIs is recommended to improve the passive range of motion or spasticity, but not to enhance active functions. The continuous posture should be applied for 1 to 2 weeks after BTIs, while adjunct active therapy has been recommended for at least 3 h per week, even though the optimal total duration is unknown. The use of low-intensity manual stretching, as well as the use of K-taping or compression sleeves, are not recommended [32].

With regard to advanced technologies, the efficacy of RAT intervention is based on the capability of inducing neuroplastic changes, rebalancing interhemispheric activation through cortical activation of the ipsilesional hemisphere, and decreasing the activation of the contralesionally hemisphere [65,66]. Some RAT may utilize the principle of paired associative stimulation (PAS) [100], which gathers electrical signals in the muscle to contract and mechanically aid the joint to move as desired without time delay. This is a self-initiated cortical stimulation that involves long-term potentiation, an important cellular mechanism for learning and memory, and induced changes in the sizes of the amplitudes of the motor-evoked potentials. These mechanisms evoked proprioceptive feedback to the cortex, resulting in plastic changes [101] and supporting the maintenance of a spasticity-improving effect in ADL, even after the effects of botulinum therapy dissipate. Previous studies have suggested that combination therapy BoNT plus RGT significantly improves muscle function in follow-up evaluations. Therefore, rehabilitation assisted by robotic devices is usually suggested to improve lower limb motor function and strength, but better effects seem related to the previous and/or concomitant use of BoNT-A.

Unfortunately, a clear recommendation on this important issue has not been reached. However, the Stroke Foundation suggested that in patients with stroke and walking difficulties, RAT (especially when combined with antispastics) could be used and may help to improve walking activities [82,89]. By now, the utility of RAGT in patients with stroke is controversial due to the lack of a consensus on standardized assessment tools or the ideal training (number of repetitions and time of therapy), frequency, and duration of the robot-assisted rehabilitation treatment to objectively evaluate the effects of RAGT.

Notably, if RAT alone may work on motor and non-motor outcomes, the combined use of robotics plus BoNT-A may further improve spasticity and related symptoms. Indeed, the decrease in muscle tone determined by the toxin may help improve the performance of a nearly physiological robotic-induced gait and/or upper limb movement.

No severe adverse events are reported with the above-mentioned therapies. However, some patients experienced pain at the point of ES application or from the casting or skin lesions caused by taping. No adverse events were reported with device-assisted training. On the other hand, the RAGT for stroke rehabilitation has several limitations. First is the lack of the generalizability of study results due to a small sample and a lack of homogenous participant groups, as well as long-term studies on efficient motor functional duration, causing a lack of a consensus rehabilitation protocol. Moreover, a clear indication of the best approach for the patients to be treated with the different robotic devices or RAGT as adjunctive therapy is not yet clear because of a lack of comparative studies. Another important point is the high costs associated with robotic technology. Cost–benefit analyses usually are not enough to justify the investment in RAGT [101,102].

We know that severe spasticity is a contraindication for the use of robotics; therefore, if a toxin may properly overcome this symptom, RAT may work better on motor functional outcomes by boosting neuroplasticity through intensive, repetitive, and task-oriented training.

Although there are differences in the modalities of training, both are based on the principles of motor learning and neuroplasticity. The response to treatment usually depends on individual variability. Some participants may respond more favorably to RAGT, while others may benefit more from traditional training methods. Therefore, individual differences are more evident as the training duration increases, with an outweighing of the cumulative effects of training [102].

This is why the previous or concomitant use of botulin toxin should be recommended in all patients with moderate to severe spasticity undergoing robotics.

## 6. Conclusions

The treatment of spasticity is a main goal in the neurorehabilitation field, considering that most patients affected by neurological disorders present with this sign and associated symptoms, including spasms and clonus. According to the available data, the combined therapy of BoNT-A with conventional or adjunct activities or robot-assisted training, especially with end-effectors, are valid tools to improve patient outcomes and performance. The combined strategies might increase BoNT-A effects, thereby lowering dosages of botulinum and reducing side effects and costs. However, better and more detailed guidelines for clinicians with practical and specific indications of outcome measures and specific RGT programs to improve patient’s goals in clinical settings are required.

## Figures and Tables

**Table 1:** The various formulations and indications of botulinum toxin.

Trade Name	Proprietary Name	Serotype	Approved Indication	Unit/Vial
Botox	OnabotulinumtoxinA	A	Blepharospasm, Hemifacial spasm, Strabismus, Cervical dystonia, Migraine, Upper limb spasticity, Lower limb spasticity (adult), Bladder, Forehead wrinkles	50, 100, 200
Xeomin	IncobotulinumtoxinA	A	Cervical dystonia, Blepharospasm, Frown lines, Upper limb spasticity, Sialorrhea in adults	100, 200
Dysport	AbobotulinumtoxinA	A	Cervical dystonia, Frown lines and wrinkles, Upper limb spasticity (adults), Lower limb spasticity (children), Lower limb spasticity (adult)	300, 500
Myobloc/Neurobloc	RimabotulinumtoxinB	B	Cervical dystonia	2000, 5000, 10,000

**Table 2:** Pharmacological treatment for spasticity.

Name	Dosing	Most Common Side Effects	Molecular Characteristics and Mechanisms of Action
Oral medications			
Baclofen	5 to 80 mg daily in divided doses (3–4 times per day) or higher for severe spasticity	Sedation, drowsiness, confusion, dizziness, weakness. Risk of withdrawal. Lowers seizure threshold	Central analog of GABA, binds presynaptic terminal GABA-B receptors and inhibits muscle stretch reflex
Tizanidine	4–36 mg (3–4 times/day)	Sedation, dizziness, hypotension, hepatotoxicity, hallucinations. Interaction with ciprofloxacine	Imidazole derivative, central a_2_- adrenergic receptor agonist in CNS
Dantrolene	100 to 400 mg (4 times/day)	Muscle weakness, sedation, gastrointestinal symptoms, hepatotoxicity	Hydantoin derivative, reduces the release of calcium from the sarcoplasmic reticulum of skeletal muscle
Diazepam	5–30 mg/d in 3–4 times/daily	Sedation, confusion, muscle weakness, gastrointestinal symptoms, memory trouble, confusion, depression, ataxia benzodiazepines may compromise neurologic recovery.	GABA-A agonist, decreases mono- and polysynaptic reflexes in the spinal cord Can be helpful to control painful muscle spasms at night.
Clonazepam	0.5–1.0 mg once daily (bedtime)	Weakness, hypotension, ataxia, discoordination, sedation, depression and memory impairment. Prolonged use could increase the risk of addiction	GABA-A agonist, decreases mono- and polysynaptic reflexes in the spinal cord Can be helpful in controlling painful muscle spasms at night.
Gabapentin	240–360 mg daily	Fainting, somnolence, nystagmus, ataxia, headache, tremor	Structurally similar to the GABA; Increases the brain level of GABA
Local Injections			
Phenol/alcohol neurolysis	Phenol concentration ≥ 3% Ethyl alcohol concentration ≥ 50%	Burning and dysesthesias. Damage of the sensory nerves and pain	
*Botulinum Toxin*	10–15 UI/kg, Intramuscular injections every 3–4 months		
Intrathecal Baclofen Therapy	Continuous intrathecal delivery, with a wide range of daily rates expressed in mg/d		

## Data Availability

Not applicable.
